# Antimicrobial nanospheres thin coatings prepared by advanced pulsed laser technique

**DOI:** 10.3762/bjnano.5.99

**Published:** 2014-06-18

**Authors:** Alina Maria Holban, Valentina Grumezescu, Alexandru Mihai Grumezescu, Bogdan Ştefan Vasile, Roxana Truşcă, Rodica Cristescu, Gabriel Socol, Florin Iordache

**Affiliations:** 1 University of Bucharest, Faculty of Biology, Microbiology Department, Aleea Portocalelor no 1–3, 060101 Bucharest, Romania; 2 University Politehnica of Bucharest, Faculty of Applied Chemistry and Materials Science, Department of Science and Engineering of Oxide Materials and Nanomaterials, Polizu Street no 1–7, 011061 Bucharest, Romania; 3 National Institute for Lasers, Plasma & Radiation Physics, Lasers Department, P.O.Box MG-36, Bucharest-Magurele, Romania; 4 S.C. Metav-CD S.A., 31 Rosetti Str., 020015 Bucharest, Romania; 5 Flow Cytometry and Cell Therapy Laboratory, Institute of Cellular Biology and Pathology “Nicolae Simionescu” (ICBP), Bucharest, Romania

**Keywords:** antimicrobial, chitosan, magnetite nanoparticles, nanospheres, *P. aeruginosa*, polylactic acid, *S. aureus*

## Abstract

We report on the fabrication of thin coatings based on polylactic acid-chitosan-magnetite-eugenol (PLA-CS-Fe_3_O_4_@EUG) nanospheres by matrix assisted pulsed laser evaporation (MAPLE). Transmission electron microscopy (TEM) and scanning electron microscopy (SEM) investigation proved that the homogenous Fe_3_O_4_@EUG nanoparticles have an average diameter of about 7 nm, while the PLA-CS-Fe_3_O_4_@EUG nanospheres diameter sizes range between 20 and 80 nm. These MAPLE-deposited coatings acted as bioactive nanosystems and exhibited a great antimicrobial effect by impairing the adherence and biofilm formation of *Staphylococcus aureus* (*S. aureus*) and *Pseudomonas aeruginosa* (*P. aeruginosa*) bacteria strains. Moreover, the obtained nano-coatings showed a good biocompatibility and facilitated the normal development of human endothelial cells. These nanosystems may be used as efficient alternatives in treating and preventing bacterial infections.

## Introduction

Driven by more and more microbial antibiotic resistance, alternative therapeutic approaches are emerging [1–4]. Polar and nonpolar, functionalized and non-functionalized magnetite nanostructures have proven successfully in combating microbial infections both in vitro and in vivo [5–6]. In the past years a series of papers have been published in prestigious journals highlighting the relevance of magnetite nanostructures in preventing the development of microbial biofilm and the opportunity of utilizing these nanosystems to obtain improved, antimicrobial coatings for biomedical applications [7–8]. Nonpolar functionalized magnetite nanostructures alone [9–10] or combined with different natural products, such as usnic acid (UA) [11] or essential oils (*Mentha piperita* [12], *Anethum graveolens* [13], *Salvia officinalis* [14], *Eugenia carryophyllata* [15]), showed improved antibiofilm effects on different types of microbial strains. Usually, these types of phyto-nano-coatings have been applied to a variety of medical surfaces in order to improve their resistance to microbial colonization [16].

Matrix assisted pulsed laser evaporation (MAPLE) processing has been applied to overcome several drawbacks of conventional solvent-based deposition techniques, such as inhomogeneous films, inaccurate placement of material, and difficult or erroneous thickness control [17–18]. MAPLE has been used to obtain thin films and coatings of soft materials, organic and polymeric materials, and complex molecules [19–35].

Furthermore, the compatibility of MAPLE processing has been demonstrated for inorganic systems such as TiO_2_ [36], and Fe_3_O_4_ nanoparticle-based materials [37], metaloporphyrines [38] and for biomolecules, e.g., poly(lactic acid) (PLA) [39], poly(lactic-*co*-glycolic acid) PLGA [40], polyvinyl alcohol (PVA) [41] and fibrinogen [42].

Our recent reports have highlighted the capability of the laser processing technique to prepare thin coatings based on polymeric microspheres. Thus, Socol et al., [43], firstly reported the novel deposition of PLGA–PVA, PLGA–PVA–BSA (bovine serum albumin) and PLGA–PVA–CS microspheres by matrix assisted pulsed laser evaporation (MAPLE) technique. SEM images of thin coatings reveal homogeneous and spherical-shaped particles in the micrometric range. The average diameter of PLGA–PVA, PLGA–PVA–BSA (bovine serum albumin) and PLGA–PVA–CS particles ranged from 180 to 250 nm. Grumezescu et al., [34], reported the MAPLE fabrication of PLA–PVA–UA microsphere thin coatings. These thin coatings possessed a homogeneous shape and showed no concavities or distortions on their surface within an average diameter of 1 μm of the deposited spheres. It is noteworthy that the microspheres maintain their initial size and do not show an aggregative behavior [34]. All these type of microspheres have been prepared by an oil-in-water emulsion-diffusion-evaporation method.

Here, we report the fabrication of thin coatings based on magnetic PLA–CS-Fe_3_O_4_@EUG nanospheres with an average diameter of the deposed spheres between 20 and 80 nm. This is the first study that reports the MAPLE processing of thin coatings containing spheres with a diameter of less than 100 nm. The thin coating is composed of nanospheres based on magnetite nanostructures and biocompatible polymers. The thin coating also exhibited antibiofilm activity, thereby opening a new perspective for the prevention of medical surfaces infections.

## Materials and Methods

### Materials

Polylactic acid (PLA), polyvinyl alcohol (PVA), chitosan (CS), eugenol (EUG), FeCl_3_, FeSO_4_·7H_2_O, NH_4_OH (25%), chloroform and *n*-hexane were purchased from Sigma-Aldrich.

#### Preparation of magnetite nanostructures

A well-known procedure described in our previous work was used to synthesize the magnetite nanostructures [44]. Briefly, EUG and NH_4_OH (25%) were added in deionized water under vigorous stirring. Then, FeCl_3_ and FeSO_4_·7H_2_O were dissolved in deionized water, and Fe^2+^/Fe^3+^ solution was dropped into the basic solution of EUG. After precipitation, magnetite–eugenol nanopowder (Fe_3_O_4_@EUG) were repeatedly washed with methanol and separated with a strong NdFeB permanent magnet.

#### Preparation of nanospheres

PLA–CS-Fe_3_O_4_@EUG nanospheres were prepared by means of a solvent evaporation method [34,45]. Thus, 4 mL PLA/chloroform solution (10 wt %) and 5 mL aqueous solution of PVA (2 wt %), CS (10 wt %) and Fe_3_O_4_@EUG (1 wt %) were emulsified with a SONIC-1200WT sonicator model from MRC for 6 min, in ON/OFF steps of 5 s and 3 s with a limitation temperature of max 40 °C, followed by solvent evaporation in 100 mL deionized water with mechanical stirring at 1000 rpm. The prepared nanospheres were thoroughly washed with deionized water and then lyophilized. PLA–CS-Fe_3_O_4_@EUG nanopheres were further used to deposit thin films by using the MAPLE technique.

#### MAPLE thin coating deposition

MAPLE targets were prepared by freezing them for 30 min at the temperature of liquid nitrogen using a suspension of 1.5% (w/v) PLA–CS-Fe_3_O_4_@EUG microspheres in *n*-hexane. The radiation of a KrF* (λ = 248 nm, τ_FWHM_ = 25 ns) COMPexPro 205 Lambda Physics-Coherent excimer laser source model impinged the frozen targets at a laser fluence of 300–500 mJ/cm^2^ and a repetition rate of 15 Hz. In order to assure the reproducibility of the nanosphere thin film deposition, the energy distribution of the laser spot was improved by using a laser beam homogenizer. During the deposition, the target was rotated with 0.4 Hz to avoid target heating and subsequent drilling. All depositions were conducted at room temperature under 0.1 Pa background pressure and a target-substrate separation distance of 4 cm by applying 45,000–160,000 subsequent laser pulses. During deposition, the MAPLE target was kept at low temperature by continuous liquid nitrogen cooling. The coatings were deposited onto glass, both sides polished (100) silicon for IRM, SEM, and biological assays. Prior to placing the substrates inside the deposition chamber, they were cleaned in an ultrasonic bath with acetone, ethanol and deionized water for 15 min, and then dried in a jet of high purity nitrogen.

### Characterization

#### Transmision electron microscopy

The transmission electron microscopy (TEM) images were obtained on finely powdered samples by using a Tecnai^TM^ G2 F30 S-TWIN high resolution transmission electron microscope manufactured by FEI Company (OR, USA). The microscope operated in transmission mode at 300 kV with a TEM point resolution of 2 Å and a line resolution of 1 Å. The prepared powder was dispersed into pure ethanol and ultrasonicated for 15 min. After that, the diluted sample was poured onto a holey carbon-coated copper grid and left to dry before TEM analysis.

#### Infrared Microscopy

IR mappings were recorded on a Nicolet iN10 MX FT-IR Microscope with an MCT liquid nitrogen cooled detector in the measurement range 4000–600 cm^−1^. Spectral collection was carried out in reﬂection mode at 4 cm^−1^ resolution. For each spectrum, 32 scans were co-added and converted to absorbance by means of the OmincPicta software (Thermo Scientiﬁc). Approximately 600 spectra were analyzed for each coating and drop cast. Four absorptions peaks known as being characteristics for the PLA–CS-Fe_3_O_4_@EUG were selected as spectral markers for the presence of nanospheres in the prepared coatings.

#### Scanning electron microscopy

SEM analysis was performed on a FEI electron microscope by using secondary electron beams with energies of 30 keV on samples covered with a thin gold layer.

### Cell viability

Human endothelial cells (EAhy926 cell line, ATCC, USA) were grown in Dulbecco's Modified Eagle Medium (DMEM) culture medium containing 10% Fetal Bovine Serum (FBS), and 1% penicillin and neomycin (Sigma Aldrich, St. Louis, MO, USA). For cell proliferation and viability CellTiter96 Non-Radioactive Cell Proliferation Assay, (Promega, Madison, USA) was used. Endothelial cells were seeded in a 96-well plate at a density of 5 × 10^3^ cells/well in DMEM medium, supplemented with 10% FBS, and incubated with nanospheres coated with eugenol for 72 h. The controls were represented by endothelial cells grown under the same culture conditions, but on bare substrates. Following the guidelines of the manufacturer the cell proliferation assay was performed in triplicates at different time intervals. Briefly, 15 µL of Promega Kit Solution I was added to each well and incubated for 4 h. Furthermore, 100 µL of Promega Kit Solution II was added to the 96-well plate and incubated for another hour. Spectrophotometry measurements were performed at 570 nm with a Mithras LB 940 spectrophotometer (Berthold Technology, Germany).

RED CMTPX fluorophore (Life Technologies, Invitrogen, USA) is a cell tracker for the long-term tracing of living cells. The RED CMTPX dye was added to the culture medium at a final concentration of 5 µM, incubated for 30 min so that the dye is able to penetrate the cells. The cells were washed with phosphate-buffered saline (PBS) and visualized by fluorescent microscopy. The nuclei were counterstained with a 1 mg/mL solution of 4',6-diamidino-2-phenylindole (DAPI). Living cells were traced in the presence of nanospheres for 5 d in culture. The micrographs were taken by a digital camera driven by the Axio-Vision 4.6 (Carl Zeiss, Germany) software.

### In vitro microbial biofilm development

*Staphylococcus aureus* ATCC 25923 and *Pseudomonas aeruginosa* ATCC 27853 strains were purchased from American Type Cell Collection (ATCC, USA). For the biofilm assays, fresh bacteria cultures were obtained in Luria Broth. Bacteria cultures were subsequently diluted as mentioned below.

The biofilm formation was assessed by using 6 multi-well plates (Nunc) in a static model for monospecific biofilm development. Coated and uncoated glass substrates were distributed in the plates containing 2 mL of microbial inoculum diluted to 10^4^–10^5^ colony forming units/mL (CFU/mL) in Luria Broth. Samples were incubated for 24 h at 37 °C. The biofilm formation was assessed after 24 h, 48 h and 72 h by a viable cell counts (VCC) assay [46].

After 24 h of incubation time, the culture medium was removed and the samples were washed with sterile PBS to remove the unattached bacteria. Coated and uncoated substrates were placed in fresh medium and incubated for an additional 24 h, 48 h and 72 h. After the incubation the samples were gently washed with sterile PBS to remove the non-adherent cells and placed in 1.5 mL micro-centrifuge tubes (Eppendorf) containing 750 μL PBS. In order to disperse biofilm cells into the suspension, the samples were vigorously mixed by vortexing for 30 s and sonicated for 10 s. Serial ten-fold dilutions were prepared and plated on Luria–Bertani (LB) agar for VCC. Experiments were performed in triplicate and repeated on three separate occasions [12,47].

### Statistical analysis

The statistical significance of the obtained results was analyzed by using GraphPad Prism version 5.04 for Windows, GraphPadSoftware, San Diego, CA, USA. For comparison, we used the number of CFU/mL as revealed by the readings of three values/experimental variants. Two-way ANOVA and Tukey’s multiple comparison tests were used for revealing significant differences among the analyzed groups.

## Results and Discussion

**Figure 1:**
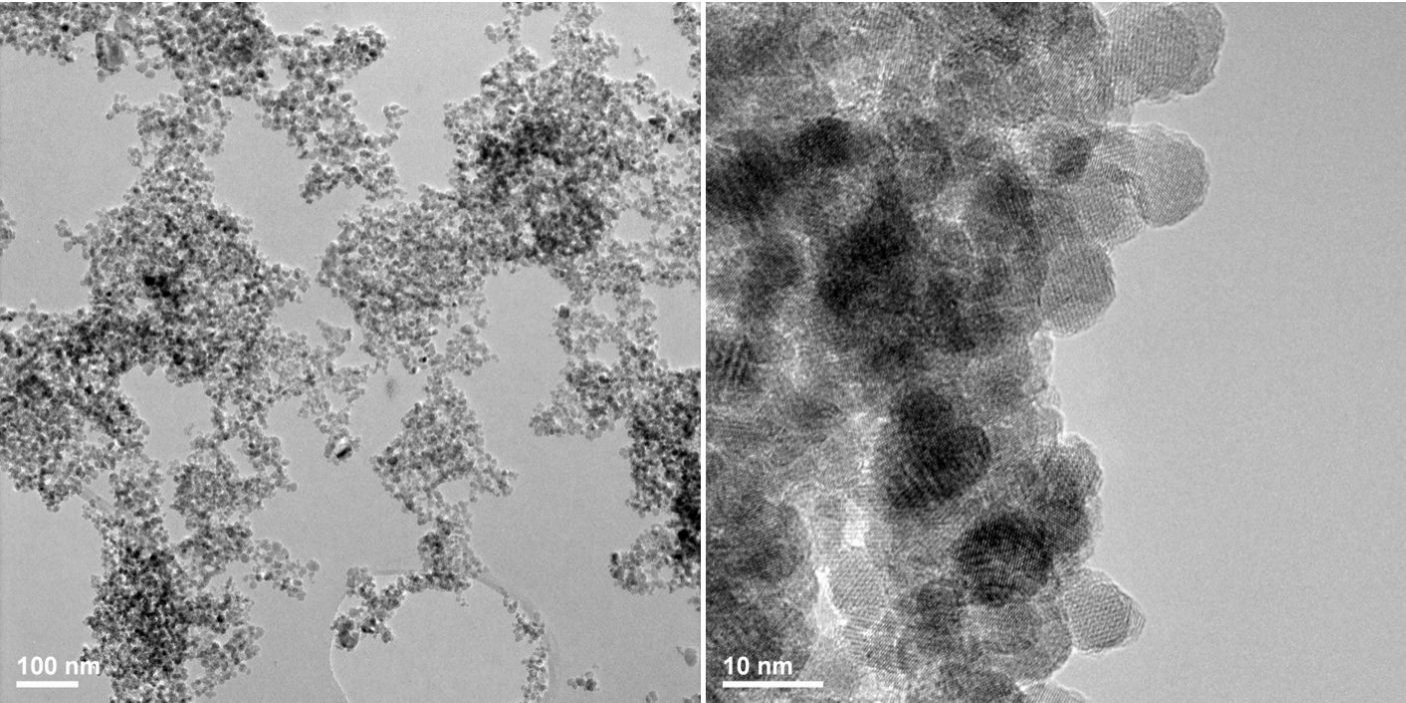
TEM images of prepared Fe_3_O_4_@EUG nanoparticles.

The morphology and size of magnetite nanoparticles was analyzed by TEM. We confirmed the nanometric dimensions of used powder in order to prepare PLA–CS-Fe_3_O_4_@EUG nanospheres. TEM images of Fe_3_O_4_@EUG at different magnification (Figure 1) show that the prepared powder has a spherical shape with a narrow size distribution of approximately 7 nm.

**Figure 2:**
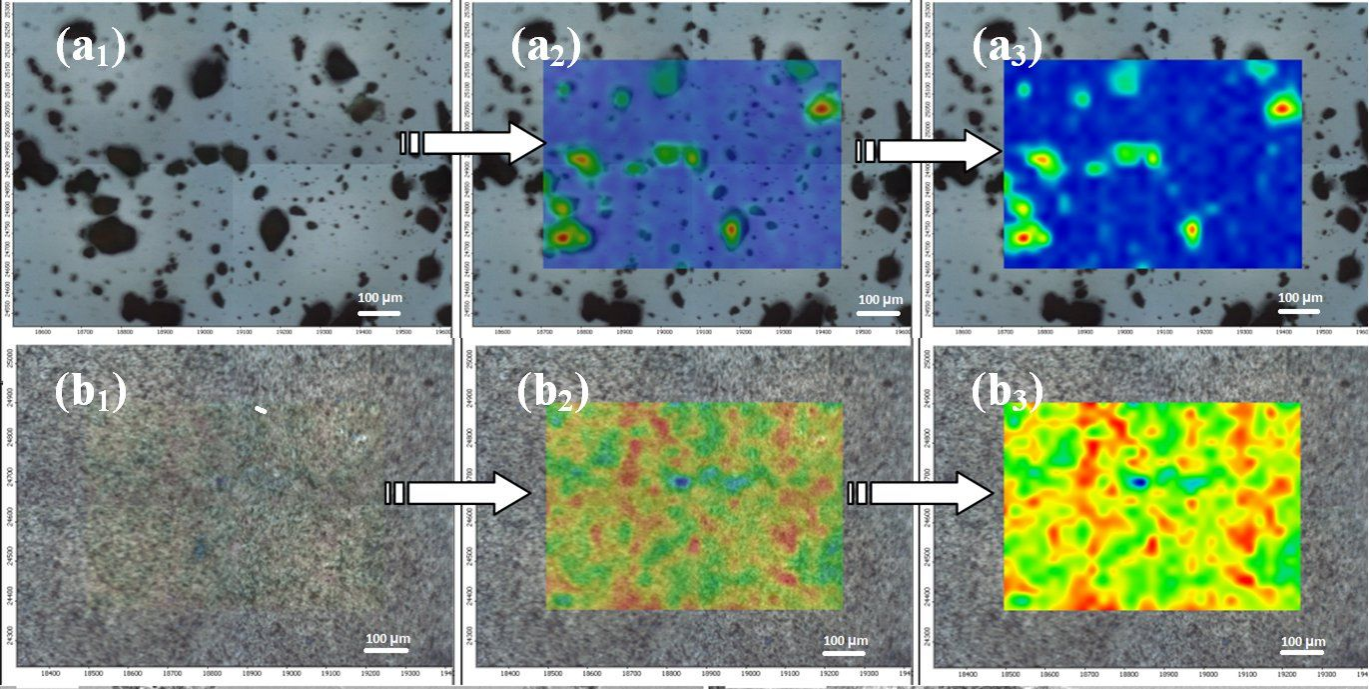
Full spectral intensity based on visible images and infrared maps of PLA-CS-Fe_3_O_4_@EUG drop cast (a) and PLA-CS-Fe_3_O_4_@EUG MAPLE thin coatings (b) overlain on the surface.

**Figure 3:**
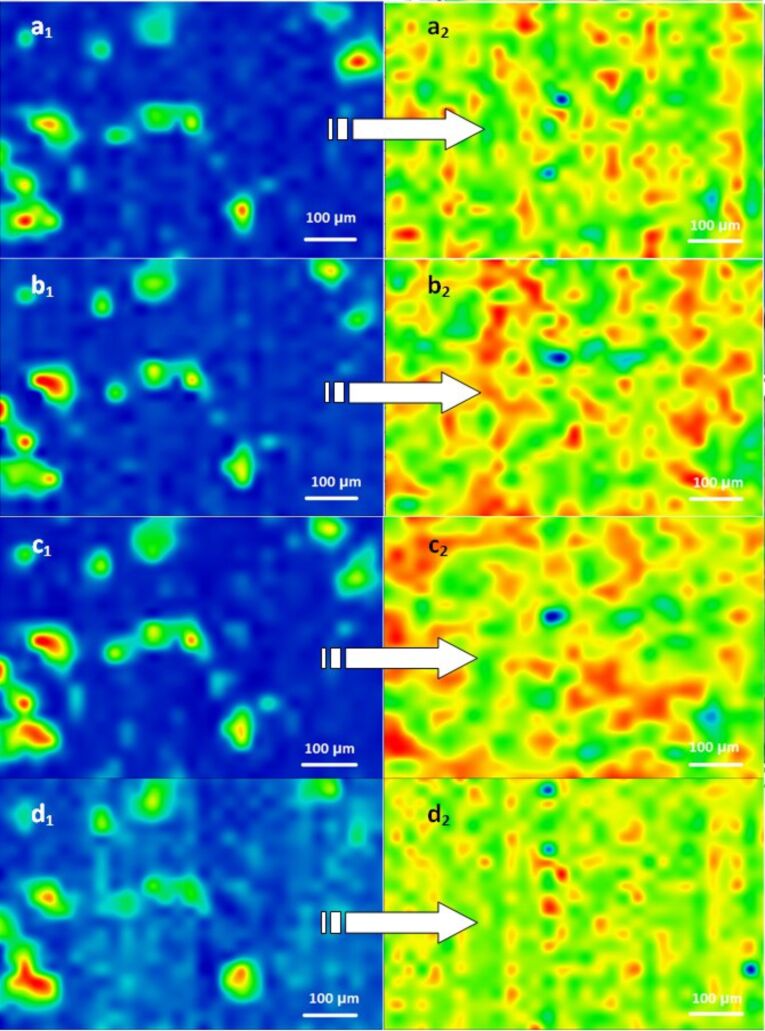
Second derivate IR mappings of the drop cast surface (1) and the thin coating (*F* = 300 mJ/cm^2^) surfaces (2). Intensity distributions are (a) 2954 cm^−1^ (CH_3_ stretch), (b) 1739 cm^−1^(C=O carbonyl group), (c) 1450 cm^−1^ (assigned to the lactides –CH_3_ group), and (d) ≈1182 cm^−1^ (–C–O– bond stretching).

Infrared microscopy was used to demonstrate the integrity of functional groups after MAPLE processing. The visible spectrum images and infrared maps based on full spectral intensity of drop cast and MAPLE thin coatings overlain on the surface are plotted in Figure 2. The prepared polymeric spheres thin coatings are distributed on the entire surface of the substrate without any free spots as can be observed on the maps of drop cast (Figure 3 a_1_, b_1_, c_1_ and d_1_).

Figure 3 shows the second derivative infrared maps of PLA–CS-Fe_3_O_4_@EUG surfaces involved in this study. Second derivative infrared mapping is used to evaluate the structural integrity of samples [42]. Absorbance intensities of IR spectra maps commensurate with the color changes starting with blue (lowest intensity) and gradually increasing through green and yellow to red (highest intensity) [43]. 600 IR spectra were analyzed for each thin coating [34].

**Figure 4:**
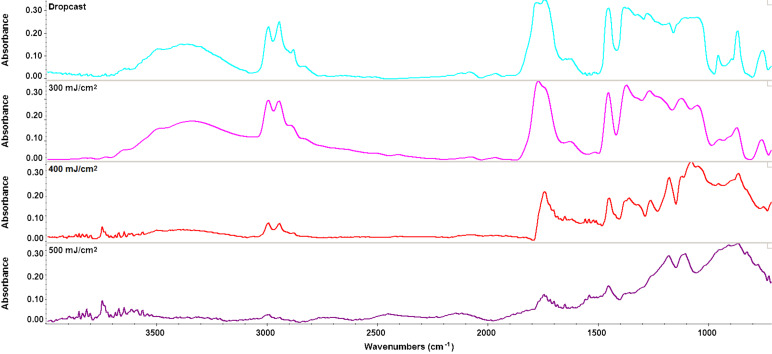
FTIR spectra of the drop cast surface and the thin coating surfaces (*F* = 300/400/500 mJ/cm^2^).

According to Figure 3 areas with moderate (green) and high intensity (red) of selected absorption bands can be observed. The tendency of nanospheres to form aggregates gives rise to the red areas. In the case of the drop cast maps, it can be concluded that there is no uniformity in the sampleand little high intensity can be observed. According to Figure 4, the thin films deposited by MAPLE (*F* = 300 mJ/cm^2^) revealed no degradation of functional groups during the laser processing.

**Figure 5:**
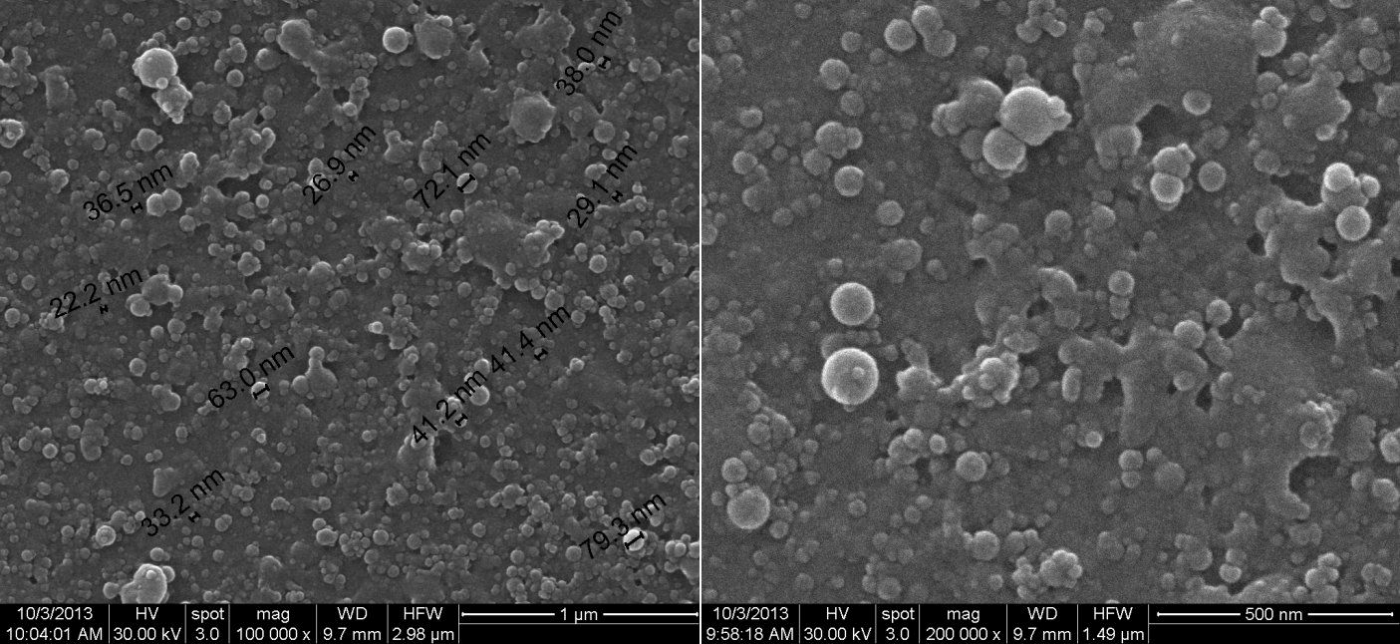
SEM images of nanosphere thin coatings prepared by MAPLE at different magnifications.

The thin coatings deposited at 300 mJ/cm^2^ laser fluence with an estimated average thickness of (≈2 μm) were analyzed by SEM (Figure 5). It can be seen that the thin coatings contain higher numbers of nanospheres on top of their surfaces with diameters between 20 and 80 nm. This is the first study that reports the MAPLE processing of thin coatings containing spheres with a diameter lower than 100 nm. Previous studies have reported the MAPLE processing of thin coatings containing spheres with diameters within the range of 180–1,000 nm [34,38].

**Figure 6:**
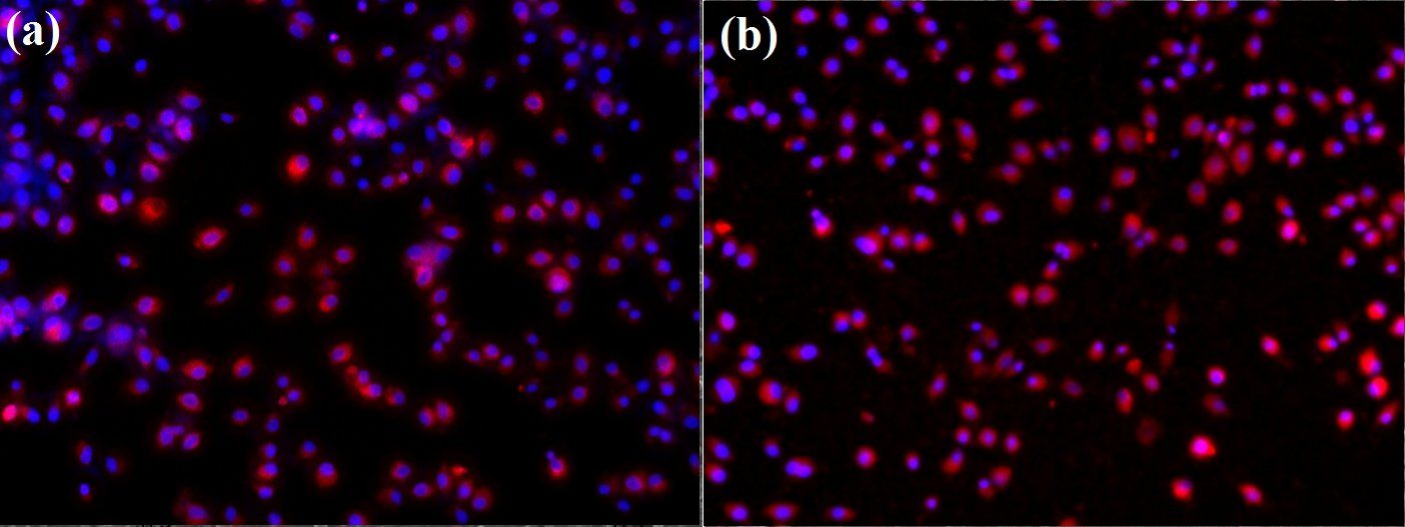
Human endothelial cells (EAhy926 cell line) after five days of growth on (a) control surface and (b) MAPLE coated surfaces.

Cytotoxicity assays revealed that the prepared nano-coatings have a great biocompatibility, and support the growth of endothelial cell cultures. The cell tracker RED CMTPX fluorophore showed that the endothelial cells are viable and exhibit a normal grow and proliferation capacity in the presence of modified nano-coated bioactive surfaces. Furthermore, the cell monolayers developed on the thin coating surfaces have a normal morphology and architecture after five days of incubation (Figure 6).

**Figure 7:**
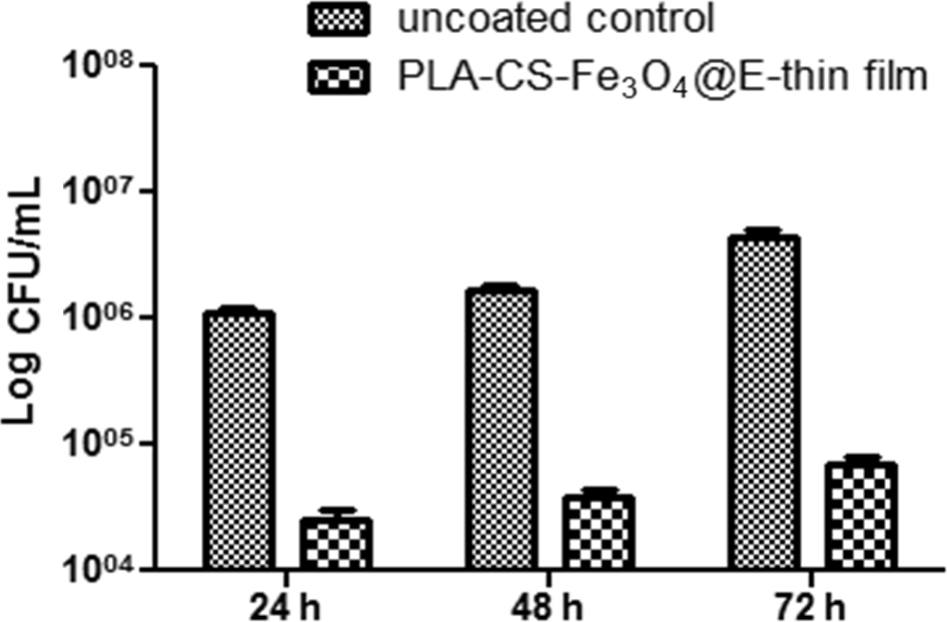
Graphic representation of viable cell count analysis after removal of *S. aureus* biofilm embedded cells 24 h, 48 h and 72 h post-infection (PLA-CS-Fe_3_O_4_@EUG thin film coatings vs uncoated control; CFU/mL = colony forming units/mL).

**Figure 8:**
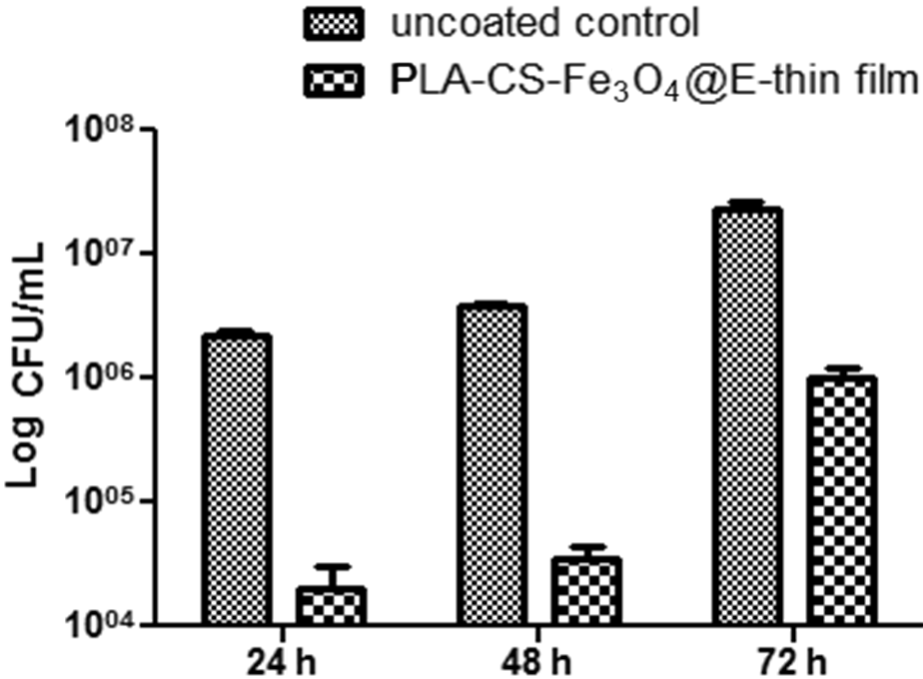
Graphic representation of viable cell count analysis after the removal of *P. aeruginosa* biofilm embedded cells 24 h, 48 h and 72 h post-infection (PLA-CS-Fe_3_O_4_@EUG thin coatings vs uncoated control; CFU/mL = colony forming units/mL).

Despite its good biocompatibility with human cells, the newly synthesized nano-active thin coating exhibited a great antimicrobial activity. The surface inhibited both *S. aureus* and *P. aeruginosa* attachment and also the formation of non-specific biofilms. MAPLE deposited thin films interfere with biofilm formation both in the initial phase and during biofilm maturation. *S. aureus* (Figure 7) biofilms were significantly impaired at all tested points of time, while *P. aeruginosa* (Figure 8) biofilms are especially affected after 24 and 48 h of incubation.

Even though magnetite nanoparticles displayed a great antimicrobial effect, many studies reported that these nanostructures may be highly toxic for hosts in higher concentrations or even active doses [48–50]. Our results demonstrate that the novel synthesized PLA–CS-Fe_3_O_4_@EUG complex nanosystems combine the proven efficacy of Fe_3_O_4_ and eugenol [40] with the biocompatibility and biodegradability of PLA and CS polymers resulting in a novel safe nanobiocomposite. Due to these characteristics PLA–CS-Fe_3_O_4_@EUG thin films represent a competitive candidate for the development of novel biomedical surfaces or devices with low costs and a high efficiency.

## Conclusion

This paper reports the successful MAPLE deposition of bioactive thin films based on PLA–CS-Fe_3_O_4_@EUG magnetic nanospheres with diameters between 20 and 80 nm. These nano-coatings displayed great antimicrobial colonization and antibiofilm formation properties, inhibiting *S. aureus* and *P. aeruginosa* biofilms. Due to the biocompatibility of this material it as a suitable candidate in developing nanostructured bioactive materials for biomedical applications.
